# Rhamnolipid-Enriched PA3 Fraction from *Pseudomonas aeruginosa* SWUC02 Primes Chili Plant Defense Against Anthracnose

**DOI:** 10.3390/ijms252312593

**Published:** 2024-11-23

**Authors:** Natthida Sudyoung, Siritron Samosorn, Kulvadee Dolsophon, Kwannan Nantavisai, Onanong Pringsulaka, Supaart Sirikantaramas, Akira Oikawa, Siriruk Sarawaneeyaruk

**Affiliations:** 1 Department of Microbiology, Faculty of Science, Srinakharinwirot University, Bangkok 10110, Thailand; natthida.sud@gmail.com (N.S.); opringsulaka@gmail.com (O.P.); 2 Department of Chemistry and Center of Excellence for Innovation in Chemistry, Faculty of Science, Srinakharinwirot University, Bangkok 10110, Thailand; siritron@g.swu.ac.th (S.S.); kulvadee@g.swu.ac.th (K.D.); 3 Department of Microbiology, Faculty of Medicine, Srinakharinwirot University, Bangkok 10110, Thailand; kwannan@g.swu.ac.th; 4 Center of Excellence in Molecular Crop, Department of Biochemistry, Faculty of Science, Chulalongkorn University, Bangkok 10330, Thailand; supaart.s@chula.ac.th; 5 Graduate School of Agriculture, Kyoto University, Kyoto 606–8502, Japan; oikawa.akira.7j@kyoto-u.ac.jp

**Keywords:** anthracnose, rhamnolipid, elicitor, biocontrol, *Pseudomonas*, *Colletotrichum*, chili plant, induced systemic resistance, transcriptome, sustainable agriculture

## Abstract

Chili anthracnose, caused by *Colletotrichum truncatum*, causes significant yield loss in chili production. In this study, we investigated the elicitor properties of a rhamnolipid (RL)-enriched PA3 fraction derived from *Pseudomonas aeruginosa* SWUC02 in inducing systemic resistance in yellow chili seedlings and antifungal activity against *C. truncatum* CFPL01 (Col). Fractionation of the ethyl acetate extract yielded 12 fractions, with PA3 demonstrating the most effective disease suppression, reducing the disease severity index to 4 ± 7.35% at 7 days post-inoculation compared with Col inoculation alone (83 ± 23.57%). PA3 also exhibited direct antifungal activity, inhibiting Col mycelial growth by 41 ± 0.96% at 200 µg/mL. Subfractionation revealed PA3 as a mixture of mono- and di-RLs, confirmed by ^1^H nuclear magnetic resonance and electrospray ionization mass spectrometry data. Additionally, PA3 enhanced seed germination and promoted plant growth without causing phytotoxicity. Transcriptomics revealed that PA3 pre-treatment prior to Col infection primed the defense response, upregulating defense-related genes involved in the phenylpropanoid, flavonoid, and jasmonic acid biosynthesis pathways, as well as those associated with cell wall reinforcement. Our findings highlight the potential of RL-enriched PA3 as both an antifungal agent and a plant defense elicitor, with transcriptome data providing new insights into defense priming and resistance pathways in chili, offering an eco-friendly solution for sustainable anthracnose management.

## 1. Introduction

Chili (*Capsicum* sp.) is cultivated worldwide, covering approximately 3.8 million hectares. India, China, Pakistan, and Thailand are among the leading chili producers [1]. Many varieties are grown for a range of culinary uses, including vegetables, spices, sauces, and condiments. Chilies are rich in vitamins, minerals, and antioxidants such as vitamin C and potassium. Capsaicin is a key bioactive compound found in chilies and is widely used in pharmaceuticals, medicines, and beverages. However, the production and quality of chilies are severely affected by pre- and post-harvest diseases caused by various pathogens such as fungi and bacteria. Among these, chili anthracnose, caused by *Colletotrichum* spp., poses a major issue, leading to yield losses of 10–80% in countries such as Thailand [2,3].

To combat diseases such as anthracnose, plants employ multilayered defense systems, including constitutive mechanisms and induced resistance such as systemic acquired resistance (SAR) and induced systemic resistance (ISR). SAR is typically triggered by pathogen infection and increases endogenous salicylic acid (SA), a crucial molecule for SAR signaling that is particularly effective against biotrophic and hemibiotrophic pathogens [4]. SAR activation leads to the expression of pathogenesis-related (PR) genes and the generation of reactive oxygen species, which together contribute to systemic resistance. PR proteins such as β-1,3-glucanase (PR-2) and chitinase (PR-3) exhibit antifungal and antibacterial activities [5]. In contrast, ISR, which is predominantly triggered by beneficial microorganisms such as plant growth-promoting bacteria (PGPB), enhances plant resistance via the jasmonic acid (JA) and ethylene (ET) signaling pathways. *Bacillus* and *Pseudomonas* are notable ISR inducers [4,6].

Elicitors are substances that activate plant defense mechanisms, triggering responses such as ISR. These elicitors can be derived from natural sources, including phytopathogens, PGPB, and plants, or be synthetically produced [7]. Novel elicitors have recently been identified and formulated as biostimulants and biocontrol products [8,9]. Using transcriptomics, researchers can comprehensively identify differentially expressed genes (DEGs) involved in defense responses, revealing how elicitors influence plant defense pathways at the molecular level. For instance, Xoca-Orozco et al. found that chitosan-treated avocado fruit exhibited more DEGs than *Colletotrichum gloeosporioides*-inoculated fruit [10]. Similarly, Decsi et al. showed that the biostimulant ELICE16INDURES^®^ enhanced soybean resistance by inducing genes related to the biosynthesis of JA, SA, phenylpropanoids, flavonoids, and phytoalexins [11]. Elicitors derived from *Pseudomonas aeruginosa* have been identified for disease control in different plants, such as 3-hydroxy-5-methoxy benzene methanol in tomatoes [8] and rhamnolipids (RLs) in rapeseed and wheat [12,13]. Given the challenges of chemical control methods for anthracnose, such as fungicide resistance and environmental risks, there is growing interest in the development of sustainable alternatives such as elicitors, supported by transcriptomics insights. Although elicitors offer a promising approach for sustainable disease management, they can also impact plant growth and yield. By activating defense pathways, elicitors trigger multipurpose intracellular signaling that initiates the synthesis of metabolites and macromolecules, potentially diverting resources from growth-related processes and inducing oxidative stress, which can hinder growth, yield, or fruit quality [12,14]. Thus, assessing their impact on overall plant growth, including seed germination, growth promotion, and fruit production, is essential to ensure that they enhance defense responses without hindering plant development.

Our previous studies demonstrated that *P*. *aeruginosa* SWUC02, a PGPB isolated from lime (*Citrus aurantifolia*), and its cell-free culture (CF-SWUC02) could effectively control canker disease in lime plants and enhance defense responses [15,16]. *P*. *aeruginosa* SWUC02 secretes protease and β-1,3-glucanase [17], which enhance its ability to inhibit fungal pathogens. Additionally, the ethyl acetate extract (eCF) from CF-SWUC02 showed the potential to protect yellow chili seedlings (*Capsicum annuum* L.) from *Colletotrichum truncatum* CFPL01 (Col) (in press) [18]. Building on these findings, the present study aimed to identify an elicitor from CF-SWUC02 to combat chili anthracnose while also assessing its effects on plant defense via transcriptomics, thereby contributing to sustainable management of chili cultivation.

## 2. Results

### 2.1. Fractionation of eCF and Its ISR Activities

**Figure 1:**
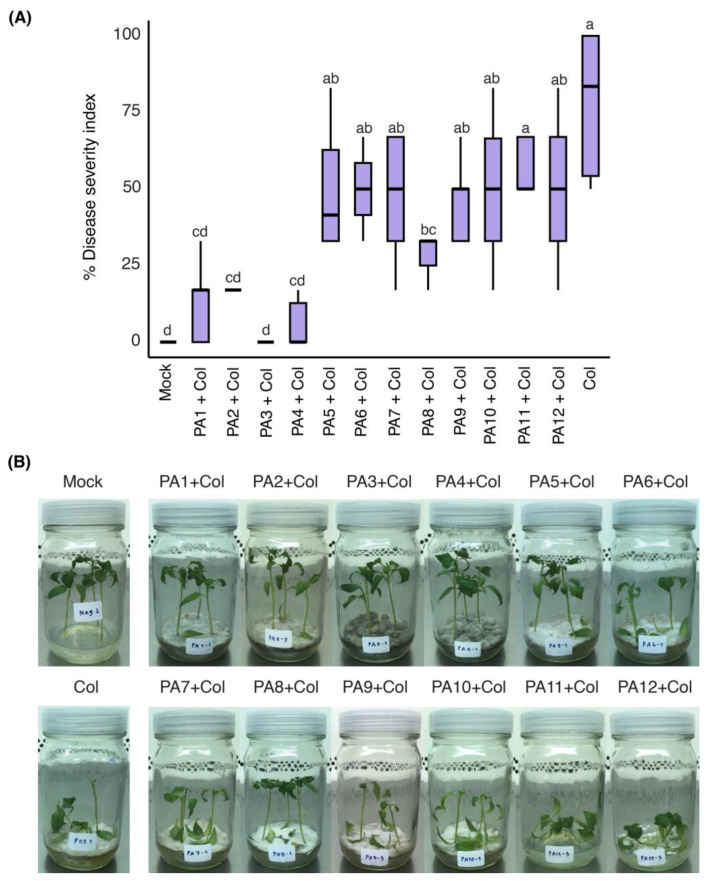
Induced systemic resistance assay of the fractions (PA1 to PA12) isolated from the ethyl acetate extract of CF-SWUC02 in yellow chili seedlings. (**A**) % DSI represented as box plots (*n* ≥ 6). Different letters (a, b, c, d) indicate significant differences (*p* < 0.05). (**B**) Disease symptoms observed on seedlings at seven dpi. Col = *C. truncatum* CFPL01.

The eCF from CF-SWUC02 was subjected to fractionation, resulting in 12 fractions (PA1-PA12) (Figure S1). Each fraction (200 µg/mL) was tested for ISR activity in yellow chili seedlings grown on Murashige and Skoog (MS) medium. The percentage of disease severity index (% DSI) at 7 days post-inoculation (dpi) is shown in Figure 1A. PA3 showed a % DSI of 4 ± 7.35, followed by PA4 (6 ± 8.61), PA1 (12 ± 12.60), PA2 (17 ± 10.54), and PA8 (31 ± 11.50), all of which were significantly lower than Col inoculation alone (83 ± 23.57). As shown in Figure 1B, the PA3 and PA4 treatments resulted in abnormal fungal colony formation on MS medium, with darkened mycelia, whereas PA11 completely inhibited Col growth but caused strong phytotoxicity, similar to PA12. Owing to its effective disease reduction and minimal impact on seedling health, PA3 was selected for further investigation.

### 2.2. Direct Antifungal Activity of Active Fraction PA3

**Figure 2:**
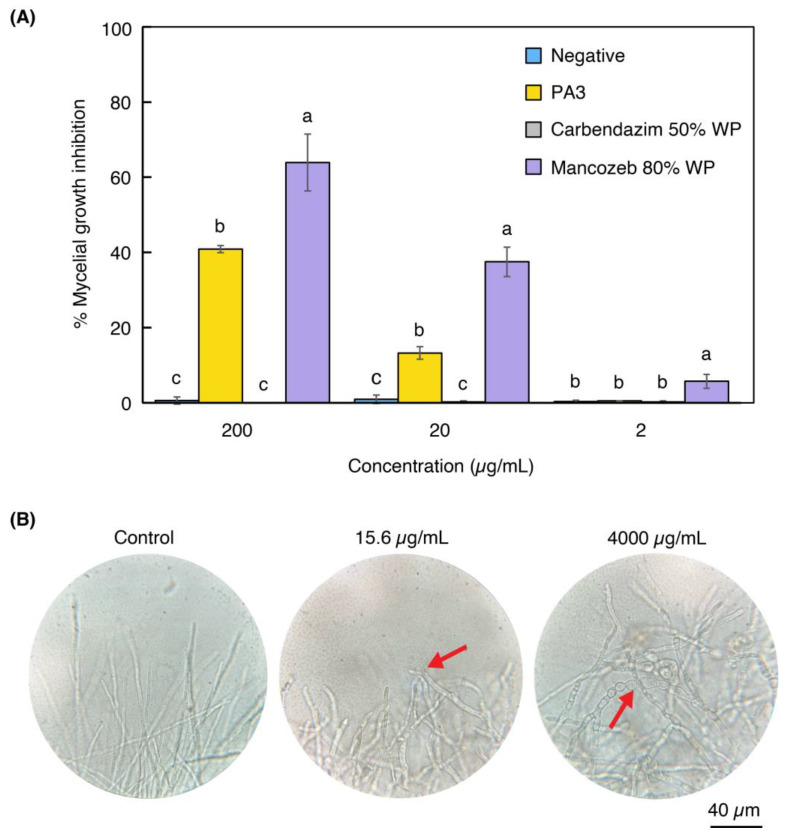
Percentage of mycelial growth inhibition (% MGI) and abnormalities in Col germination under PA3 treatment. (**A**) % MGI by PA3, carbendazim 50% WP, and mancozeb 80% WP at 200, 20, and 2 µg/mL. The negative control was potato dextrose agar without any supplements. Bars represent mean ± SD. Difference letters (a, b, c) indicate significant differences (*p* < 0.05) between treatments within each concentration. (**B**) Microscopic observations (1000×) of germinated hyphae from conidia treated with PA3 at 0 (control), 15.6, and 4000 µg/mL. Red arrows highlight abnormalities.

The PA3 fraction exhibited antifungal activity against Col at concentrations of 200 and 20 µg/mL, inhibiting mycelial growth by 41 ± 0.96% and 13 ± 1.66%, respectively. While mancozeb 80% wettable powder (WP) demonstrated a stronger inhibitory effect, with 64 ± 7.54%, 38 ± 3.92%, and 6 ± 1.85% inhibition at 200, 20, and 2 µg/mL, respectively, carbendazim 50% WP exhibited no antifungal activity at any of the tested concentrations (Figure 2A). Additionally, light microscopy examination revealed that PA3 induced morphological abnormalities in Col mycelia, such as branching at low concentrations (15.6 to 125 µg/mL), while higher concentrations (250 to 4000 µg/mL) resulted in more severe effects, including swollen hyphae and vacuolation (Figure 2B and Figure S2). These results highlight the potential of PA3 as a strong antifungal agent, making it a promising alternative to conventional fungicides.

### 2.3. Identification of Elicitor Compound(s)

**Figure 3:**
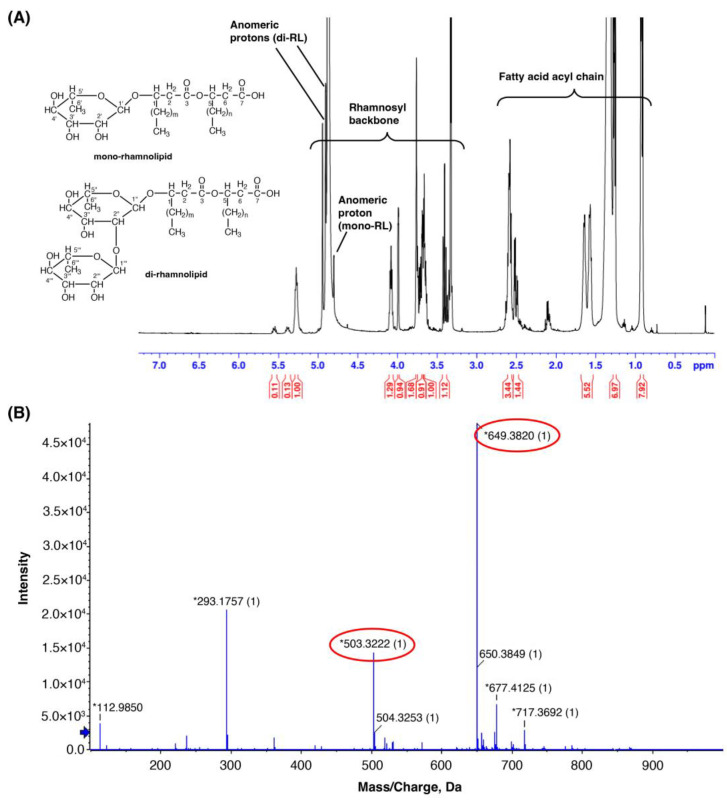
PA3 characteristic by ^1^H NMR and ES-MS spectroscopic data. (**A**) ^1^H NMR spectrum of PA3 in CD_3_OD, showing characteristic proton signals with the chemical structure of mono- and di-RLs displayed. (**B**) Negative-ion electrospray mass spectrum of PA3, with red circles highlighting the main detected peaks corresponding to molecular ions of mono- and di-RLs. The blue arrow indicates the detection limit, while the asterisks indicate the calculated monoisotopic masses of the detected ions.

The chemical composition of the promising PA3 fraction was determined using silica gel thin-layer chromatography (TLC) (Figure S1A). The TLC analysis indicated the PA3 fraction as a mixture of two components: PA3-1 as the major component (lower R_f_) and PA3-2 as the minor component (higher R_f_). Further characterization of the mixture was performed using nuclear magnetic resonance (NMR) spectroscopy and electrospray ionization mass spectrometry (ES-MS). The PA3 fraction consisted of a mixture of di-RL (Rha-Rha-C_10_-C_10_; PA3-1) and mono-RL (Rha-C_10_-C_10_; PA3-2) (Figure 3). The ^1^H NMR spectrum (Figure 3A) revealed characteristic lipid backbone signals detected at 0.8–1.0 ppm as a triplet (methyl protons), 1.30–2.65 ppm as a broad singlet (α-methylene protons adjacent to the carbonyl group), and 4.10 and 5.30 ppm as two triplets (β-methine protons adjacent to the carbonyl group). The rhamnosyl backbone signals were observed at 1.20 ppm as two doublets (methyl protons), 3.35–4.00 ppm as a multiplet (methine protons), and 4.90 ppm as a doublet (anomeric protons). Moreover, negative-ion electrospray analysis revealed two main peaks [M–H]^−^ at *m*/*z* 503.3222 and 649.3820, where M represents the molecular weights of Rha-C_10_-C_10_ and Rha-Rha-C_10_-C_10_, respectively (Figure 3B).

**Figure 4:**
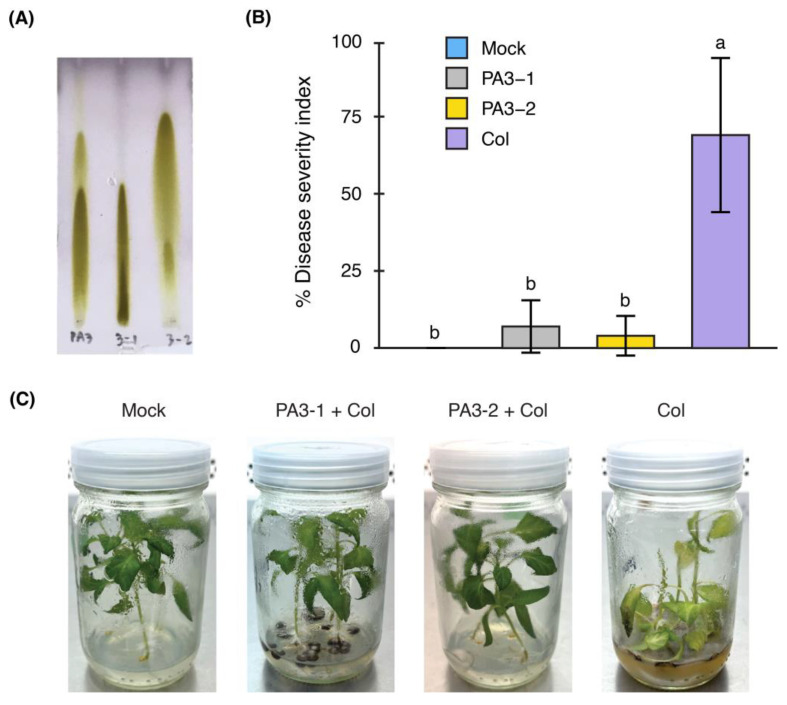
Characterization of PA3 subfractions (PA3-1 and PA3-2) and their effects on disease symptoms in yellow chili seedlings. (**A**) Thin-layer chromatography (TLC) plate analysis of PA3 (**left**), PA3-1 (**middle**), and PA3-2 (**right**), visualized with *p*-anisaldehyde staining. (**B**) Percentage disease severity index (% DSI) of seedlings in mock, PA3-1, PA3-2, and Col treatments. Bars represent mean ± SD. Different letters (a, b) indicate significant differences (*p* < 0.05). (**C**) Disease severity symptoms observed on seedlings across different treatments.

To identify the specific ISR elicitor role of each RL in the PA3 fraction, PA3-1 and PA3-2 in the mixture were separated using preparative TLC on silica gel to give PA3-1 and PA3-2 in the ratio of 1.7:1. The subfractions were validated by analytical TLC and visualized using *p*-anisaldehyde staining (Figure 4A). The structures of purified PA3-1 and PA 3-2 were further confirmed using ^1^H NMR and ES-MS data (Figures S3 and S4) and compared to previously reported data [19,20].

The effects of PA3-1 and PA3-2 on disease symptoms in yellow chili seedlings were tested. Both subfractions (200 µg/mL each) significantly reduced the severity of Col infection in the seedlings, as indicated by a significant decrease in % DSI compared to the Col inoculation alone (69 ± 24.97%). Specifically, PA3-1 reduced % DSI to 7 ± 8.49%, and PA3-2 to 4 ± 6.42% (Figure 4B). Figure 4C illustrates the visible reduction in disease symptoms in seedlings treated with both subfractions.

### 2.4. Effect of PA3 on Seed Germination and Plant Growth Promotion

**Figure 5:**
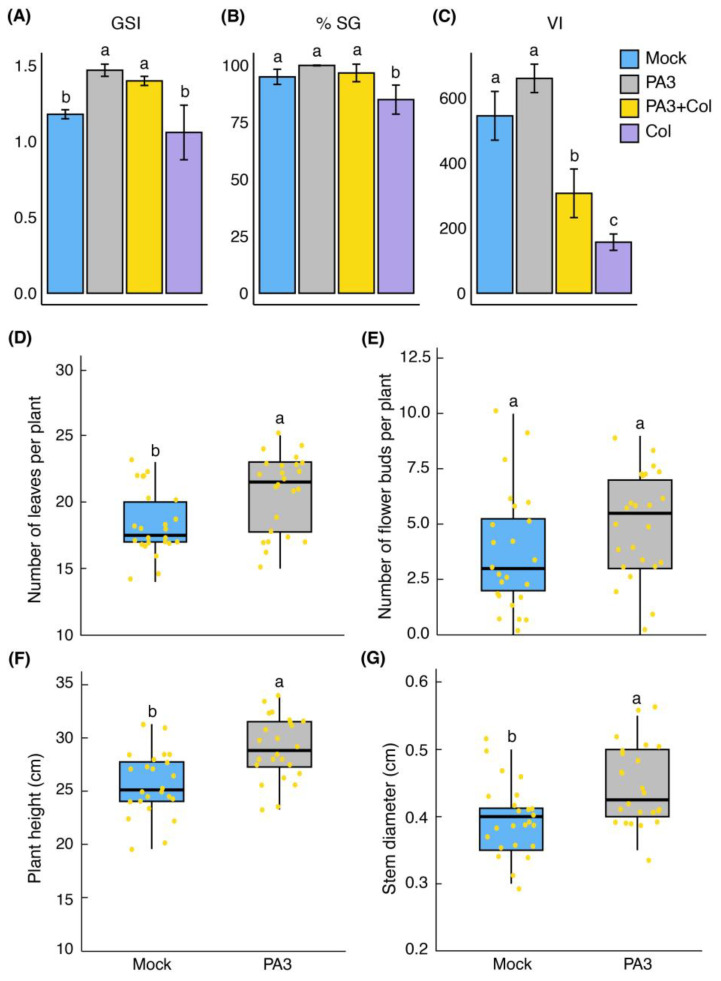
Effects of PA3 application on seed germination and plant growth of yellow chili. (**A**) Germination speed index (GSI). (**B**) Percentage of seed germination (% SG). (**C**) Vigor index (VI). (**D**) Number of leaves per plant. (**E**) Number of flower buds per plant. (**F**) Plant height (cm). (**G**) Stem diameter (cm). Each dot represents an individual sample. Different letters (a, b, c) indicate statistically significant differences between treatments (*p* < 0.05).

Col infection alone significantly reduced germination, with a seed germination percentage (% SG) of 85.0 ± 6.38 and a seedling vigor index (VI) of 156.9 ± 25.13, which were the lowest values observed. However, pre-treatment with PA3 before Col inoculation improved these parameters, showing a higher germination speed index (GSI) of 1.40 ± 0.03, % SG of 96.7 ± 3.85, and VI of 307.2 ± 74.76. PA3 treatment alone had no adverse effects on seed germination or seedling performance compared to the control (mock) treatment. Notably, PA3 treatment, with or without Col inoculation, led to an increase in the GSI compared to the mock treatment (Figure 5A–C).

In the pot experiment, PA3 treatment significantly promoted vegetative growth, resulting in an increased number of leaves (20.67 ± 2.90), plant height (29.02 ± 2.96 cm), and stem diameter (0.44 ± 0.06 cm) compared to the mock treatment (Figure 5D–G). However, the positive effects of PA3 on the fruit development parameters were not statistically significant (Table S1). Overall, these results indicate that PA3 enhances seed germination, promotes seedling growth, and protects against Col infection without causing phytotoxicity.

### 2.5. Transcriptome Analysis Reveals Synergistic Defense Responses in Yellow Chili Seedlings Following PA3 Pre-Treatment and Col Infection

The raw sequence data generated by the transcriptome analysis were submitted to the NCBI Sequence Read Archive (BioProject PRJNA1141217, BioSample SAMN42883329 to SAMN42883340). The total number of bases per sample ranged from 3.2 to 3.6 Gb, with consistently high-quality sequencing results (Table S2). After trimming, 98% of the reads were retained, with a Q20 value of approximately 99% (Table S3). De novo assembly generated 509,029 transcripts, with a GC content of 41% and an N50 of 1661 bases (Table S4). BUSCO analysis revealed 94% transcriptome completeness, with 85% of the reads aligned to the transcriptome. Clustering at 95% identity grouped the sequences into 453,463 clusters and identified 110,132 complete open reading frames.

**Figure 6:**
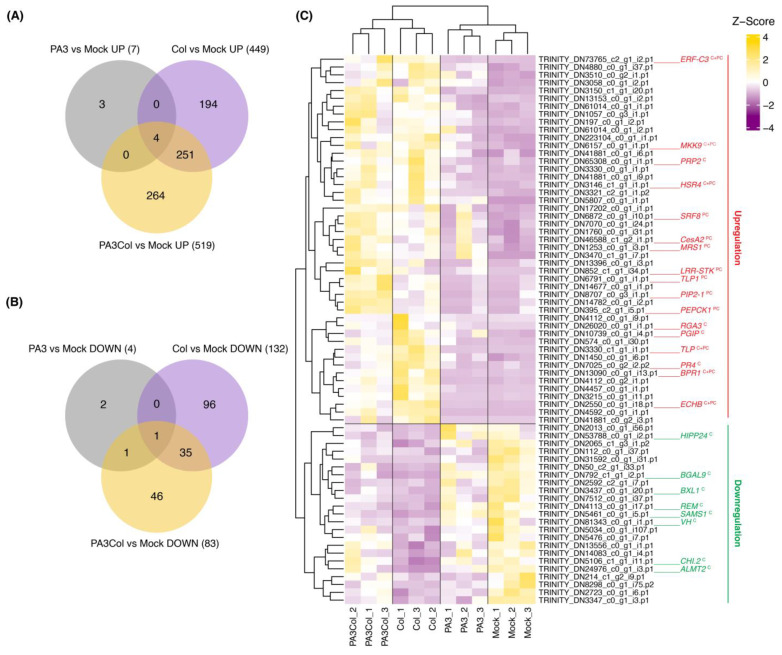
Venn diagrams and heatmap of DEGs related to defense response. (**A**) Venn diagram showing the number of upregulated DEGs. (**B**) Venn diagram showing the number of downregulated DEGs. (**C**) Heatmap of 70 defense-related DEGs identified by GO terms, displaying Z-scores of gene expression levels. Genes highlighted in red indicate upregulated genes, while those in green indicate downregulated genes. Superscripts indicate the following: C = significant DEGs in Col treatment alone, PC = significant DEGs in PA3+Col treatment, and C + PC = significant DEGs in both treatments.

Principal component analysis (PCA) demonstrated distinct expression patterns across treatments. The Col and PA3+Col treatments were clearly separated from the mock treatment, whereas PA3 alone partially overlapped with all groups (Figure S5). DEG analysis revealed that PA3 treatment alone elicited minimal changes, resulting in 11 DEGs, with both upregulated and downregulated genes (Figure 6A,B, and Table S5). Notably, one upregulated DEG (false discovery rate [FDR] < 0.05) was the cytokinin riboside 5′-monophosphate phosphoribohydrolase LOG1 gene, involved in cytokinin activation [21], which likely plays a role in promoting vegetative growth. In contrast, Col infection alone led to 581 DEGs, whereas the combination of PA3 and Col resulted in 602 DEGs (Figure 6A,B, and Table S5). Hierarchical clustering of 70 DEGs (FDR < 0.05) related to plant defense, identified by Gene Ontology (GO) terms such as defense response to fungus (GO:0050832) and plant-type cell wall (GO:0009505) (Table S6), showed unique expression patterns for the Col and PA3+Col treatments (Figure 6C). Among the selected DEGs, no significant difference in expression was observed between PA3 and the mock treatments.

Several upregulated DEGs involved in plant defense mechanisms were significantly elevated in both Col and PA3+Col treatments (represented by the red text with superscript C+PC in Figure 6C). These included ethylene-response factor C3 (*ERF-C3*), mitogen-activated protein kinase kinase 9 (*MKK9*), and hypersensitivity-related 4-like (*HSR4*) (FDR < 0.01). PR genes, such as endochitinase B (*ECHB*), thaumatin-like protein (*TLP*), and basic PR protein 1 (*BPR1*) (FDR < 0.001), were also elevated in both treatments, indicating the antimicrobial responses of the plants. In contrast, some PR genes, such as *PRP2* (FDR < 0.01), the putative disease resistance protein RGA3 (FDR < 0.05), and PR-4-like (*PR4*) (FDR < 0.001), along with a polygalacturonase inhibitor (*PGIP*) (FDR < 0.001), were significantly upregulated only in the Col treatment group (represented by the red text with superscript C in Figure 6C), indicating a pathogen-specific response. However, the PA3+Col treatment activated a broader defense response. Genes such as thaumatin-like protein 1 (*TLP1*) (FDR < 0.001), aquaporin PIP2-1 (FDR < 0.01), phosphoenolpyruvate carboxykinase 1 (*PEPCK1*), protein strubbelig-receptor family 8 (*SRF8*), and probable LRR receptor-like serine/threonine-protein kinase (*LRR-STK*) (FDR < 0.05) were uniquely upregulated. Moreover, genes involved in cell wall reinforcement, such as cellulose synthase A catalytic subunit 2 (*CesA2*) and mannan synthesis-related 1 (*MRS1*) (FDR < 0.05), were significantly upregulated only upon PA3+Col treatment (represented by the red text with superscript PC in Figure 6C).

In addition, the downregulated DEGs involved in plant defense mechanisms were significantly reduced during Col infection alone (represented by the green text with superscript C in Figure 6C). Genes responsible for maintaining cell wall integrity, such as β-galactosidase 9 (*BGAL9*) and β-D-xylosidase 1 (*BXL1*) (FDR < 0.01), were suppressed. A regulator of biotic stress response, remorin (*REM*) (FDR < 0.01), was also downregulated. Enzymes involved in the biosynthesis of defense-related compounds, including S-adenosylmethionine synthase 1 (*SAMS1*), chalcone isomerase (*CHI.2*) (FDR < 0.01), and vicianin hydrolase (*VH*) (FDR < 0.05), showed reduced expression. Furthermore, genes related to metal stress tolerance, such as heavy metal-associated isoprenylated plant protein 24 (*HIPP24*) (FDR < 0.05) and aluminum-activated malate transporter 2 (*ALMT2*) (FDR < 0.01), were suppressed. However, PA3 pre-treatment reduced the suppressive effects of Col infection on these downregulated DEGs.

**Figure 7:**
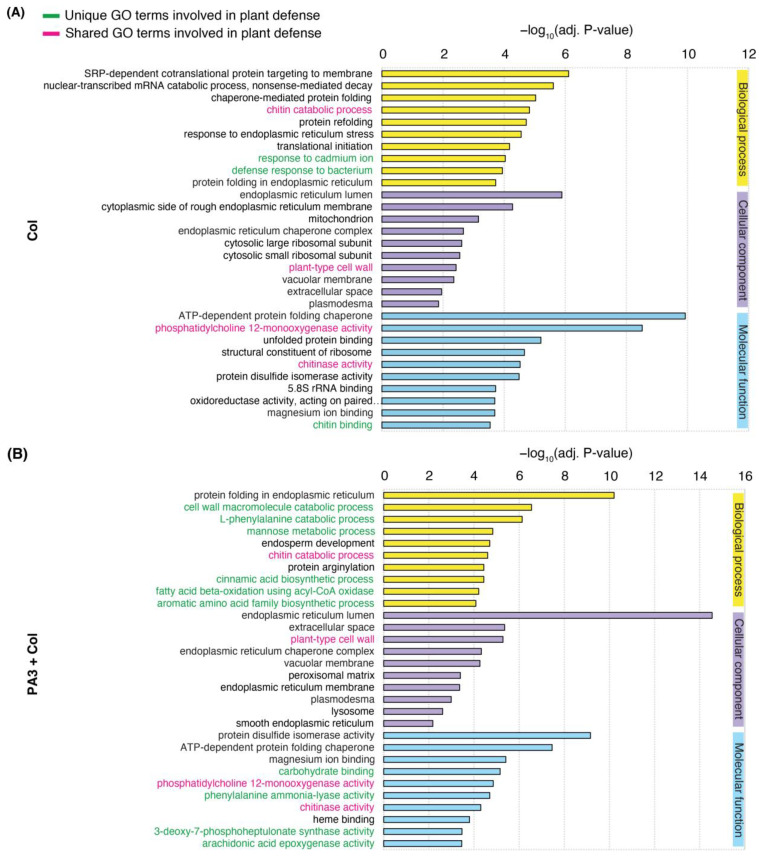
Top 10 enriched GO terms across three categories—biological process (yellow), cellular component (purple), and molecular function (blue)—related to DEGs. (**A**) Enriched GO terms in the Col treatment alone. (**B**) Enriched GO terms in the PA3+Col treatment. Unique defense-related GO terms are indicated in green, while shared defense-related GO terms between treatments are highlighted in pink.

GO term enrichment analysis (adj. *p* < 0.05) further revealed distinct biological responses across the treatments. The PA3 treatment alone had a minimal impact, with no significant GO terms identified owing to the low number of DEGs. Both the Col and PA3+Col treatments resulted in shared and unique defense-related processes among the top 10 GO terms in each category: biological process, cellular component, and molecular function. The shared GO terms indicated common defense strategies between the Col and PA3+Col treatments, whereas the unique GO terms highlighted treatment-specific responses (Figure 7). Among the shared terms were functions such as chitin catabolic process (GO:0006032), plant-type cell wall (GO:0009505), and chitinase activity (GO:0004568). Despite these similarities, Col treatment alone highlighted unique terms associated with stress, as evidenced by GO terms enriched in response to endoplasmic reticulum stress (GO:0034976), protein refolding (GO:0042026), structural constituent of ribosome (GO:0003735), response to cadmium ion (GO:0046686), defense response to bacterium (GO:0042742), and chitin binding (GO:0008061) (Figure 7A). In contrast, the PA3+Col treatment showed unique terms related to broader metabolic and defense mechanisms, such as the cell wall macromolecule catabolic process (GO:0016998), cinnamic acid biosynthesis process (GO:0009800), aromatic amino acid family biosynthetic process (GO:0009073), phenylalanine ammonia-lyase activity (GO:0045548), and mannose metabolic process (GO:0006013) (Figure 7B). These findings suggest that PA3 enhances and modulates the defense responses of yellow chili plants during Col infection.

### 2.6. Metabolic Pathway Analysis Highlights Enhanced Defense Activation as PA3 Primes Response to Resist Col Attack

A Kyoto Encyclopedia of Genes and Genomes (KEGG) pathway analysis revealed key insights into the pathways influenced by Col and PA3+Col treatments. Col infection alone influenced 31 pathways, including ribosome, citrate cycle, glutathione metabolism, and cysteine and methionine metabolism (Figure S6A), whereas PA3+Col treatment significantly affected 25 pathways, including other glycan degradation, arachidonic acid metabolism, steroid hormone biosynthesis, biosynthesis of various alkaloids, and tryptophan metabolism (Figure S6B). Both treatments shared enriched pathways related to defense mechanisms, such as phenylpropanoid biosynthesis, flavonoid biosynthesis, and α-linolenic acid metabolism.

**Figure 8:**
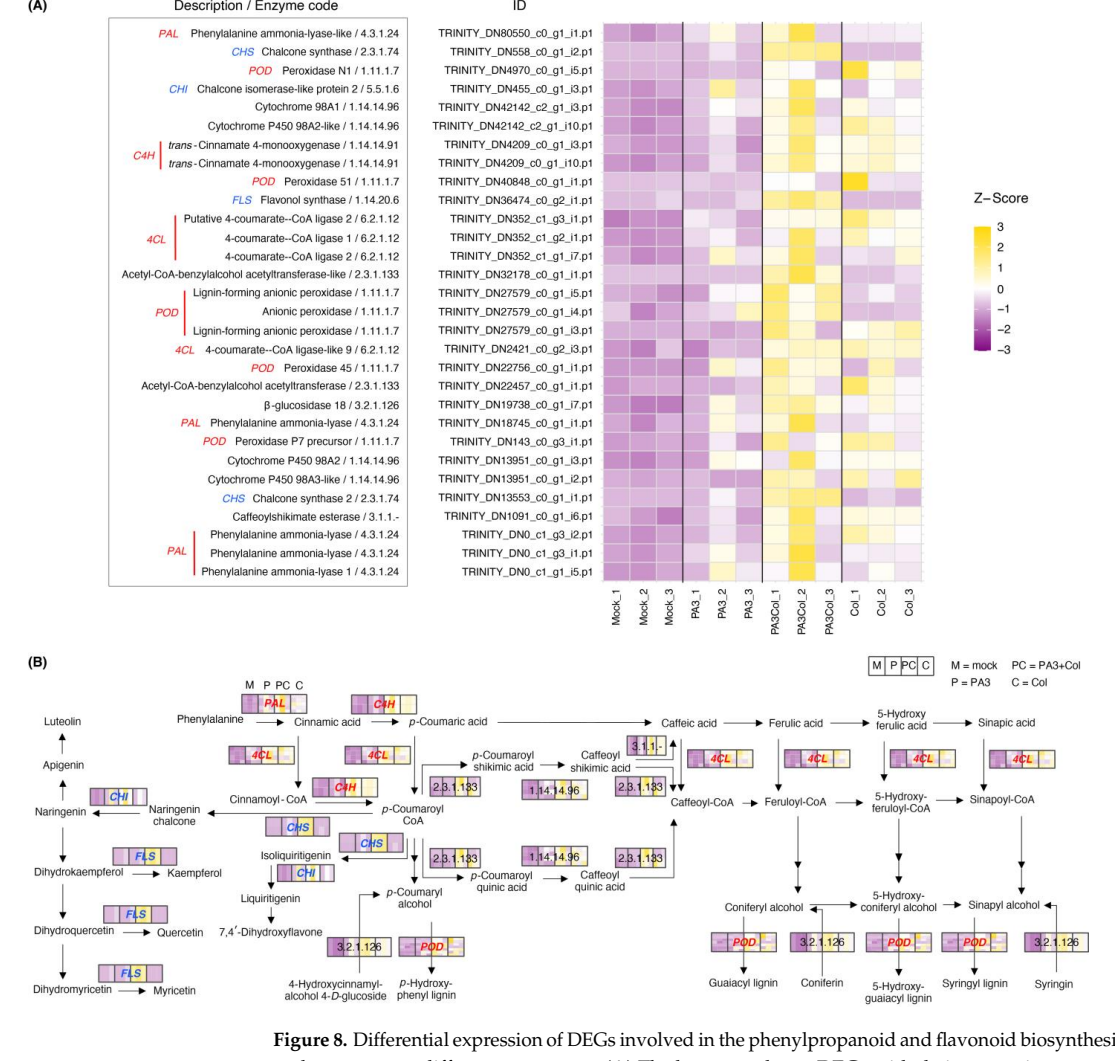
Differential expression of DEGs involved in the phenylpropanoid and flavonoid biosynthesis pathways across different treatments. (**A**) The heatmap shows DEGs with their respective enzyme codes and gene IDs, with gene expression levels represented as Z-scores, indicating relative expression changes compared to the mean expression across treatments. (**B**) The schematic diagram illustrates the phenylpropanoid and flavonoid biosynthesis pathways. The colors within the boxes correspond to the Z-scores in the heatmap. Treatments are abbreviated as M = mock, P = PA3, PC = PA3+Col, and C = Col. DEGs encoding enzymes involved in the phenylpropanoid pathway are shown in red text, while those involved in the flavonoid pathway are shown in blue text.

The PA3+Col treatment showed a more pronounced effect on the DEGs involved in phenylpropanoid and flavonoid biosynthesis (Figure 8 and Table S7). Notably, PA3+Col led to a strong upregulation of the gene encoding phenylalanine ammonia-lyase (*PAL*; 4.3.1.24), the first enzyme in the phenylpropanoid pathway that converts phenylalanine to *trans*-cinnamic acid. Additional upregulated genes included the enzymes *trans*-cinnamate-4-monooxygenase (*C4H*; 1.14.14.91), which converts cinnamic acid to *p*-coumaric acid, and 4-coumarate–CoA ligase (*4CL*; 6.2.1.12), which produces CoA esters such as *p*-coumaroyl CoA and caffeoyl CoA. Furthermore, peroxidase (*POD*; 1.11.1.7), which is involved in lignin biosynthesis, was significantly upregulated. While Col treatment produced similar effects, the expression levels were lower than those of the combined treatment. PA3 alone induced a slight, non-significant increase relative to the mock treatment. Notably, genes involved in flavonoid biosynthesis, such as chalcone synthase (*CHS*; 2.3.1.74), chalcone isomerase-like protein 2 (*CHI*; 5.5.1.6), and flavonol synthase (*FLS*; 1.14.20.6) were significantly upregulated in the PA3+Col treatment, potentially contributing to the accumulation of flavonoids such as apigenin, luteolin, and quercetin.

**Figure 9:**
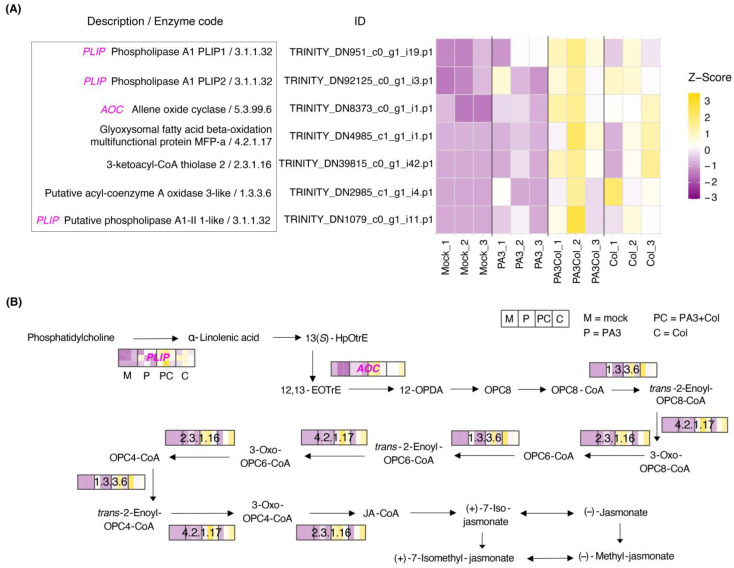
Differential expression of DEGs involved in the α-linolenic acid biosynthesis pathway across different treatments. (**A**) The heatmap shows DEGs with their respective enzyme codes and gene IDs, with gene expression levels represented as Z-scores, indicating relative expression changes compared to the mean expression across treatments. (**B**) The schematic diagram illustrates the α-linolenic acid biosynthesis pathway, with enzyme codes and DEGs mapped to specific reactions. Treatments are abbreviated as M = mock, P = PA3, PC = PA3+Col, and C = Col.

Furthermore, we observed upregulation of genes involved in JA biosynthesis within the α-linolenic acid metabolism pathway, particularly with the PA3+Col and Col treatments (Figure 9 and Table S8). Plastid lipases (*PLIP*; 3.1.1.32), which release α-linolenic acid from galactolipids and phospholipids, and allene oxide cyclase (*AOC*; 5.3.99.6), which converts 12,13-epoxy-9(*Z*),15(*Z*)-octadecatrienoic acid (12,13-EOTrE) to *cis*-(+)-12-oxo-phytodienoic acid (12-OPDA), were upregulated. Subsequent transformations of 12-OPDA to JA involved acyl-coenzyme A oxidase 3-like (1.3.3.6), glyoxysomal fatty acid β-oxidation multifunctional protein MFP-a (4.2.1.17), and 3-ketoacyl-CoA thiolase (2.3.1.16).

Overall, the combined PA3+Col treatment most effectively activated plant defense mechanisms by upregulating genes in both the phenylpropanoid/flavonoid and JA biosynthesis pathways, suggesting that pre-treatment with PA3 could enhance plant disease resistance.

### 2.7. Validation of Transcriptomics Data Using Reverse Transcription Quantitative PCR (RT-qPCR)

**Figure 10:**
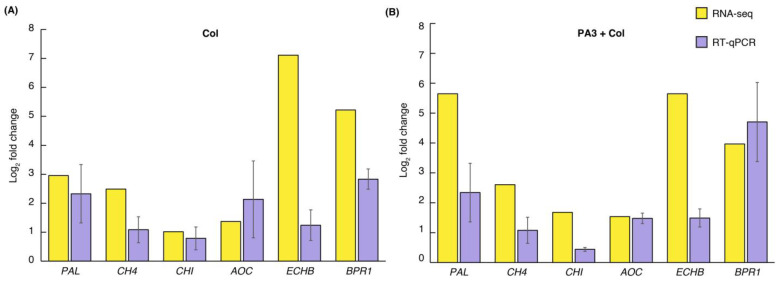
Validation of gene expression levels by RNA-seq and RT-qPCR for six DEGs: *PAL*, *C4H*, *CHI*, *AOC*, *ECHB*, and *BPR1*. (**A**) Fold change comparison in the Col treatment alone relative to the mock treatment; and (**B**) fold change comparison in the PA3+Col treatment relative to the mock treatment. Yellow bars represent RNA-seq results, while purple bars represent RT-qPCR results, shown as log_2_ fold change.

Six upregulated DEGs from both the PA3+Col and Col treatments, including genes from the phenylpropanoid (*PAL*, *C4H*), flavonoid (*CHI*), and α-linolenic acid (*AOC*) pathways, and two defense-related genes (*ECHB*, *BPR1*), were validated using RT-qPCR. Figure 10 provides a comparative analysis of the RNA-seq and RT-qPCR data, revealing higher fold changes in RNA-seq but with consistent expression patterns. These findings corroborate the reliability and accuracy of the transcriptomic data.

## 3. Discussion

Further purification and analysis of the eCF revealed that the PA3 fraction (200 µg/mL) exhibited the strongest ability to elicit plant resistance against anthracnose while being non-toxic to seedlings. Although Col colonies were detected in the medium, they were morphologically altered, suggesting a direct antifungal effect of PA3. Fractions PA1, PA2, and PA4 showed reduced ISR-eliciting activity, potentially containing the same active compounds as PA3, though in lower concentrations. In contrast, PA8, which similarly reduced % DSI, may contain different bioactive compounds. Khan et al. identified antifungal compounds such as 3,9-dimethoxypterocarpan, pyochelin, quinolones, and cascaroside B in the eCF of *P. aeruginosa* Ld-08 [22], whereas Fatima and Anjum reported that 3-hydroxy-5-methoxy benzene methanol from *P. aeruginosa* PM12 exhibited ISR-eliciting properties against Fusarium wilt [8]. In our study, PA3 inhibited Col mycelial growth more effectively than carbendazim, a fungicide commonly used in Thailand to control chili anthracnose, but was less effective than mancozeb. Carbendazim, a systemic fungicide, disrupts cell division by inhibiting microtubule synthesis, rendering it susceptible to resistance. In contrast, mancozeb, a contact fungicide, inhibits multiple enzyme systems and reduces the resistance potential [23].

Using ^1^H NMR and ES-MS analyses, we identified mono-RL (Rha-C_10_-C_10_) and di-RL (Rha-Rha-C_10_-C_10_) as the key active components of PA3. Both the RLs are common congeners produced by *P. aeruginosa* [12,24]. Mono-RLs contain one rhamnose unit, whereas di-RLs contain two units, with the rhamnose providing hydrophilic properties and fatty acid hydrophobicity. This amphiphilic structure enables RLs to reduce surface tension, facilitating their widespread applications in detergents, food, medicine, cosmetics, and agriculture. RLs enhance soil quality, promote plant growth, and suppress plant pathogens [25]. Lahkar et al. explored the antifungal properties of a crude biosurfactant of RLs produced by *P. aeruginosa* JS29 and demonstrated its efficacy as a biofungicide against *C. truncatum*, with both preventive and curative effects on anthracnose in red chili plants [26]. RLs also act as plant elicitors, inducing plant resistance to pathogens [12,13,27]. Given the increasing resistance of *C. truncatum* to conventional fungicides [23], exploring alternative solutions such as integrated pest management or the use of elicitors, such as RLs, may help manage resistance and protect chili crops. The mode of action of RLs involves damage to microbial cell membranes, which minimizes the likelihood of pathogen resistance. This compromises cell integrity, resulting in leakage of cellular contents, vacuolation, and swollen branched hyphae [28,29], which ultimately lead to cell death and hinder the ability of the fungi to infect chili seedlings [26]. Our findings are consistent with these observations, revealing that RLs offer two benefits in sustainable agriculture: direct antifungal action and stimulation of defense mechanisms in yellow chili. This suggests that RL-enriched PA3 can be broadly applied to various *C. annuum* varieties, expanding their potential for managing anthracnose in different chili varieties.

Moreover, germination assays demonstrated that PA3 significantly enhanced yellow chili seed germination and increased resistance to Col. Furthermore, pot experiments revealed that PA3 treatment promoted chili plant growth, potentially by improving nutrient uptake and root function. These findings are consistent with those of Sancheti et al., who reported that RLs improved soybean seed imbibition and water uptake, leading to faster germination. RLs at concentrations of 500–1000 µg/mL can also stimulate lateral root development [30]. Additionally, the well-established antifungal properties of RLs likely increased germination rates and improved seedling health by mitigating the effects of Col infection.

As previously mentioned, RLs function not only as antimicrobial agents but also act as microbe-associated molecular pattern (MAMP) elicitors, triggering plant defense responses through signaling pathways such as SA and JA/ET [12]. Although RLs have been previously studied as plant elicitors, knowledge of their roles in chili plants (*C. annuum*) against *C. truncatum* is limited. This study fills this gap by employing transcriptomics to provide a comprehensive view of RLs as elicitors in chili plants. Most studies investigating the elicitor properties of RLs have primarily relied on basic methods such as evaluating plant defense enzymes or gene expression; however, recent advancements in omics technologies offer a more comprehensive approach. For example, Pierre et al. utilized a comparative quantitative proteomic analysis to gain a deeper understanding of how RLs trigger defense responses in rapeseed plants. They suggested that RLs activate both local and systemic defense responses by influencing proteins involved in plant defense, secondary metabolism, and signaling pathways [31].

In the transcriptome analysis, we observed that PA3 alone had a minimal impact on chili seedlings, as evident by the transcriptome profile, suggesting a neutral fitness cost at the tested concentration. This finding aligns with studies showing that RLs prime plant immunity without causing major adverse effects. For example, RLs protected rapeseed from *Botrytis cinerea* through both antifungal activity and defense activation, including reactive oxygen species production, defense gene expression, callose deposition, and stomatal closure, without impairing growth or chlorophyll content [12]. Similarly, RLs provided protection to wheat against *Zymoseptoria tritici* by direct antifungal action and transient early regulation of defense genes, all while maintaining plant metabolic balance [13]. Col infection alone triggered the upregulation of defense-related genes, including those encoding PR proteins (endochitinases pcht28, P2, endo-1,3-β-glucosidase, PR-4-like) and the polygalacturonase inhibitor. These genes are components of the specific defense pathways that plants employ to combat Col. PR proteins such as endochitinase and glucanase directly target fungal cell walls to inhibit pathogen growth and play crucial roles in SAR [32]. The upregulation of the polygalacturonase inhibitor suggests that plants produce proteins to inhibit enzymes degrading pectin, a key component of plant cell walls, thereby restricting pathogen invasion [33]. Notably, the higher number of upregulated DEGs upon PA3+Col treatment highlights their synergistic effect, activating additional defense pathways and metabolic processes that were not prominent in the individual treatments. Additionally, this treatment also resulted in fewer downregulated DEGs than Col alone, suggesting that PA3 reduces the negative effects of Col, maintaining cellular homeostasis, and reducing stress. By priming the defense system, PA3 facilitates ISR, allowing for a rapid and robust response when Col is introduced. The upregulated DEGs, such as *TLP1*, *PEPCK1, PIP2-1*, *SRF8*, *LRR-STK*, *CesA2*, and *MSR1*, suggest a broad defense response involving antifungal defense, stress response, enhanced signaling, and cell wall fortification [32,34,35,36,37,38]. PA3 primes plant defenses and reduces the Col population, leading to a weaker pathogen-specific response than Col treatment alone.

PR genes, such as *BPR1* and *ECHB*, were upregulated in both treatments, indicating shared defense mechanisms. *BPR1* overexpression is known to enhance tolerance to heavy metal stress and pathogens by modulating redox systems and stress-response genes [39], whereas increased *ECHB* expression in *Nicotiana benthamiana* boosts resistance against bacterial pathogens [40]. Additionally, *MKK9* and *ERF-C3*, which are involved in ET biosynthesis and signaling, were elevated under both treatments. Slavokhotova et al. observed strong ET signaling induction in response to *Cucumber green mottle mosaic virus* at the early infection stage, suggesting that enhanced ET biosynthesis and signaling contribute to biotic stress responses, which is consistent with our findings [41].

The downregulation of DEGs involved in plant defense during Col infection indicated a weakening of defense mechanisms, particularly via the suppression of genes associated with plant cell wall integrity (*BGAL9* and *BXL1*) [42,43], biotic stress (*REM*) [44], and defense compound biosynthesis (*SAMS1*, *CHI.2*, *VH*) [45,46,47,48]. Additionally, the reduced expression of metal stress tolerance genes (*HIPP24* and *ALMT2*) suggests heightened susceptibility to abiotic stress [49,50]. However, PA3 pre-treatment effectively reduced these suppressive effects by restoring the expression of defense-related genes.

The top 10 unique GO terms from the Col treatment alone could be grouped into three categories: protein maintenance and quality control (e.g., response to protein refolding), protein synthesis and regulation (e.g., translational initiation), and a broader defense response, including the defense response to bacteria. In contrast, GO terms enriched in the PA3+Col treatment, such as the L-phenylalanine catabolic process, cinnamic acid biosynthesis, and aromatic amino acid family biosynthetic process, highlighted the enhanced role of the phenylpropanoid pathway in producing antimicrobial compounds and strengthening cell walls [8]. Additionally, the enrichment of the GO term fatty acid β-oxidation using acyl-CoA oxidase suggests its involvement in boosting JA biosynthesis for ISR activation [47].

KEGG pathway analysis revealed that PA3 alone did not significantly affect metabolic pathways, whereas PA3+Col treatment significantly affected multiple pathways. Enhanced other glycan degradation suggests an increased breakdown of complex carbohydrates, whereas biosynthesis of various alkaloids indicates enhanced production of antimicrobial alkaloids. Arachidonic acid metabolism is involved in signaling pathways that regulate defense responses, whereas tryptophan metabolism is key for synthesizing defense-related compounds such as indole alkaloids. This combined treatment induced a broader range of metabolic processes than Col treatment alone. However, both PA3+Col and Col treatments showed enriched pathways, including phenylpropanoid and flavonoid biosynthesis and α-linolenic acid metabolism, indicating shared defense mechanisms in plants [51]. Consistent with previous findings [51,52,53,54], the combined treatment resulted in the most pronounced upregulation of defense-related genes, including *PAL*, *C4H*, and *4CL*, in the phenylpropanoid pathway, which is essential for the production of various defense compounds. Additionally, the genes encoding *POD*, which is involved in lignin biosynthesis, were upregulated. In the flavonoid pathway, genes such as *CHS*, *CHI*, and *FLS* were significantly upregulated with the PA3+Col treatment (more pronounced than with Col alone), potentially leading to the accumulation of flavonoids such as apigenin and luteolin, which are related involved in plant defense. Moreover, the combined treatment resulted in the upregulation of genes involved in JA biosynthesis, such as *PLIP* and *AOC*, within the α-linolenic acid metabolism pathway. As JA is a crucial signaling molecule in ISR, it is vital in regulating stress responses and development [47].

Overall, our findings highlight the priming effect of PA3 prior to Col challenge, leading to a more robust and comprehensive defense response than the individual treatments. Therefore, RL-enriched PA3 acts as an elicitor in chili plants, priming defense pathways that enhance both pathogen resistance and overall stress tolerance. Although this study focused on *C. truncatum* in chili, RLs have been reported to trigger defenses in various plants and act as biofungicides [12,13,31], suggesting the RL-enriched PA3 fraction may have applications beyond this crop and pathogen.

Future research should explore the practical applications of PA3 in agricultural settings by optimizing field application strategies, with attention to concentration, timing, and method. Additionally, investigating the effects of PA3 on a broader range of crops and pathogens could extend its potential as an eco-friendly tool for integrated disease management.

## 4. Materials and Methods

### 4.1. Plant and Microorganisms

Chili plants (*C. annuum* variety Leung Ampan, East-West Seed, Nonthaburi, Thailand) and the fungal pathogen *C. truncatum* CFPL01 (Col), isolated from infected chili fruit, were used. The pathogen was previously identified using the internal transcribed spacer region, glyceraldehyde-3-phosphate dehydrogenase gene, and β-tubulin gene (accession numbers OQ996551, OR018988, and OR018990, respectively) (in press) [18]. *P. aeruginosa* SWUC02 was cultured in tryptone soya broth (HiMedia, Thane, India) with 0.01% (*w*/*v*) CuCl_2_ at 30 °C and 150 rpm for 6 days. After centrifugation, CF-SWUC02 was collected and stored at −20 °C for subsequent experiments [15].

### 4.2. Extraction, Fractionation, and Identification of Elicitor Compounds from CF-SWUC02

CF-SWUC02 was subjected to extraction twice with ethyl acetate. The eCF was evaporated and further fractionated using size-exclusion chromatography on a Sephadex™ LH 20 column (Cytiva, Uppsala, Sweden) and eluted with methanol. The fractions were analyzed by TLC on silica gel plates (Merck, Darmstadt, Germany) using a mixture of dichloromethane and methanol (95:5, *v*/*v*) as the mobile phase. Fractions with similar TLC patterns were pooled and tested for ISR activity. The active fraction PA3 (43.4 mg), exhibiting the strongest ISR activity, was further separated into subfractions using multiple development preparative TLC on silica gel (5% methanol in dichloromethane) to give PA3-1 (26.3 mg) and PA3-2 (15.3 mg), both as brown viscous liquids. The main components of the active subfractions were characterized using ^1^H NMR (500 MHz Bruker Avance, Bruker, Bremen, Germany) and ES-MS spectroscopy (AB Sciex Triple TOF 6600, AB SCIEX, Concord, ON, Canada).

### 4.3. ISR Assay

The ISR potential of the extracts against Col was evaluated by assessing their ability to reduce the % DSI in six-leaf yellow chili seedlings cultivated under tissue culture conditions. Each tested solution (3 mL at 200 µg/mL) was applied to the surface of the MS plant medium (PhytoTech Labs, St. Lenexa, KS, USA). For fraction testing, 10% (*v*/*v*) dimethyl sulfoxide (DMSO) was used as the solvent, whereas for PA3 and its subfractions, sterile distilled water (SDW) was used. The treated seedlings were incubated for 48 h at 22 ± 2 °C. Subsequently, a conidial suspension (10^6^ conidia/mL) of Col was swabbed onto two fully expanded leaves per seedling. Mock and positive controls were treated with either 10% (*v*/*v*) DMSO or SDW instead of the tested solution. In the second treatment, a sterile 0.05% (*v*/*v*) Tween 80 solution was applied to the mock-treated seedlings, whereas the positive control seedlings were swabbed with the conidial suspension. Disease symptoms were evaluated at 7 dpi using the severity scores provided in Table S9, and % DSI was calculated using the formula described by Yang et al. [55]. Statistical analysis was performed using the Kruskal–Wallis and Dunn’s tests (*p* < 0.05).

### 4.4. Antifungal Assay of Active Fraction PA3

The antifungal activity of the PA3 active fraction was tested against Col and compared with that of carbendazim 50% WP (Thaion Chemical, Bangkok, Thailand) and mancozeb 80% WP (Sotus home and garden, Nonthaburi, Thailand). A poisoned food assay was performed in a 6-well plate, with each well containing 2 mL of potato dextrose agar (HiMedia, Thane, India) mixed with either PA3 or the fungicides at final concentrations of 200, 20, or 2 µg/mL in SDW. A fungal plug was placed in the center of each well and incubated for 3 days before calculating the percentage of mycelial growth inhibition (% MGI), as described by Toghueo et al. [56]. Statistical analysis was performed using the Kruskal–Wallis and Dunn’s tests (*p* < 0.05). In contrast, the morphology of the germinated hyphae from Col conidia incubated for 24 h at varying PA3 concentrations (4000 to 7.8 µg/mL) was observed using light microscopy (Zeiss, Oberkochen, Germany) (1000×) and compared to a negative control where conidia germinated in potato dextrose broth (HiMedia, Thane, India).

### 4.5. Evaluation of PA3 Effects on Seed Germination and Plant Growth

#### 4.5.1. Seed Germination

Chili seed germination and early seedling growth were assessed under four treatments: mock, PA3, Col, and PA3+Col. Sixty seeds per treatment were subjected to surface sterilization, soaked in either PA3 solution (200 µg/mL) or SDW for 30 min, and then air-dried. The seeds were immersed in a conidial suspension of Col or a sterile 0.05% (*v*/*v*) Tween 80 solution, placed on sterile moistened filter papers, and incubated in the dark at 30 °C for 10 days. GSI, % SG, and VI were calculated as described by Rajabi et al. [57]. Statistical analysis was performed using the Kruskal–Wallis and Dunn’s tests (*p* < 0.05).

#### 4.5.2. Plant Growth and Fruit Development

Two-month-old yellow chili plants were grown in a 2:1 (*v*/*v*) soil–sand mixture. Plants were drenched with either 20 mL of the PA3 solution (200 µg/mL) or SDW alone three times at 7-day intervals. After one month, growth parameters were measured, including leaf number, flower bud number, height (cm), and diameter (cm). Additionally, fruit characteristics, including fruit length and width and fresh and dry weight, were recorded over the next two months. Statistical analysis was performed using the Mann–Whitney U test (*p* < 0.05).

### 4.6. Transcriptome Analysis

#### 4.6.1. Seedling Treatment

Six-leaf-stage yellow chili seedlings grown under sterile conditions were divided into four treatments: mock, PA3, Col, and PA3+Col. The PA3 group was treated with a PA3 solution (200 µg/mL), while the Col group was inoculated with a conidial suspension (10^6^ conidia/mL). The PA3+Col group received the PA3 treatment, followed by Col inoculation after 48 h. PA3 was added to the MS medium, and Col inoculation was performed by swabbing the conidia onto the leaves. The mock group was treated with SDW, followed by a sterile 0.05% (*v*/*v*) Tween 80 solution without PA3 treatment or Col inoculation.

#### 4.6.2. RNA Extraction and Transcriptome Analysis

Total RNA was extracted from leaves 48 h post-inoculation using the FavorPrep™ Plant Total RNA Mini Kit (Favorgen, Ping Tung, Taiwan), with DNase I treatment (Invitrogen, Waltham, MA, USA). Twelve high-quality samples (RNA Integrity Numbers > 7), representing three replicates from each of the four treatments, were subjected to sequencing on an Illumina NovaSeq X platform (Illumina, San Diego, CA, USA) using 150-bp paired-end reads at Biomarker Technologies Co., Ltd. (Beijing, China).

A bioinformatics pipeline was used to identify DEGs in response to treatments using the OmicsBox software (v3.1.11) (BioBam, Valencia, Spain). Quality assessment was performed using FastQC (v0.11.8) and low-quality reads were removed using Trimmomatic (v0.38). The cleaned reads were assembled de novo using Trinity (v2.15.1), and transcriptome completeness was assessed using BUSCO (v5.4.5) against the Solanales OrthoDB v10 database. Sequence redundancy was minimized using CD-HIT (v4.8.1) and coding regions were predicted using TransDecoder (v5.7.1). The predicted coding regions were annotated using Diamond BLASTx (v2.1.9) against the RefSeq Non-Redundant database (9 January 2024) and further annotated using GO mapping, InterProScan, and EggNOG, as implemented within OmicsBox (v3.1.11), for comprehensive functional insights. GO terms were assigned to all annotated genes and filtered using the chili pepper taxonomy (*C. annuum*; taxon ID: 4072). Transcript-level quantification was performed using RSEM (v1.3.3), and normalization was performed using the gene-length-corrected trimmed mean of M-values method [58]. Differential expression analysis was conducted using edgeR (v3.18) to identify significantly altered genes with a log_2_|FC| >1 and FDR < 0.05. These DEGs were further analyzed for biological significance through GO term and KEGG pathway enrichment analyses using Fisher’s exact test (adjusted *p* < 0.05).

#### 4.6.3. Validation of Transcriptome Data

One-step RT-qPCR was conducted on six DEGs, *PAL*, *C4H*, *CHI*, *AOC*, *ECHB*, and *BPR1*, using the KAPA SYBR FAST One-Step Universal Kit (Kapa Biosystems, Wilmington, MA, USA) on a CFX96 Touch Real-Time PCR system (Bio-Rad, Hercules, CA, USA). Specific primers were designed using the NCBI Primer-BLAST (Table S10). Fold changes were calculated using the 2^−ΔΔCT^ method [59]. The E3 ubiquitin-protein ligase (*E3UB*) gene, selected based on its stable expression across all samples and previously reported as a reference gene [60], was used for normalization. Data were analyzed by one-way ANOVA with a least significant difference (LSD) test (*p* < 0.05).

## 5. Conclusions

This study represents the first transcriptome analysis to explore the potential of microbial RLs produced by *P. aeruginosa* SWUC02 as elicitors in chili plants against anthracnose caused by *C. truncatum*. The PA3 fraction, which is rich in mono- and di-RLs, displayed notable antifungal activity, inhibited mycelial growth, and caused structural abnormalities in the fungal mycelia. PA3 not only inhibits fungal growth but also acts as an elicitor, priming the plant defense system by upregulating key pathways, including phenylpropanoid, flavonoid, and JA biosynthesis. This priming effect enhances plant resistance, leading to a more integrated and robust defense response against pathogen infection. Additionally, PA3 improved seed germination and promoted vegetative growth without negatively affecting plant growth. These findings emphasize the potential of PA3 as a component of an eco-friendly integrated disease management strategy for chili cultivation. Future research should focus on optimizing the application of PA3 to maximize its efficacy in the field and explore its potential to protect other crops against fungal pathogens.

## Data Availability

The original contributions presented in the study are included in the article/Supplementary Material, further inquiries can be directed to the corresponding author.

## Supplementary Materials

The supporting information can be downloaded at: https://www.mdpi.com/article/10.3390/ijms252312593/s1.
