# Supplementary material for: Rhamnolipid-Enriched PA3 Fraction from Pseudomonas aeruginosa SWUC02 Primes Chili Plant Defense Against Anthracnose
Source: Int J Mol Sci. 2024 Nov 23;25(23):12593. doi: 10.3390/ijms252312593 (PMC11641530; doi:10.3390/ijms252312593)
Supplement: Supplementary file 1 [file ijms-25-12593-s001.zip › Supplementary_Figures.pdf]

(A)

(B)

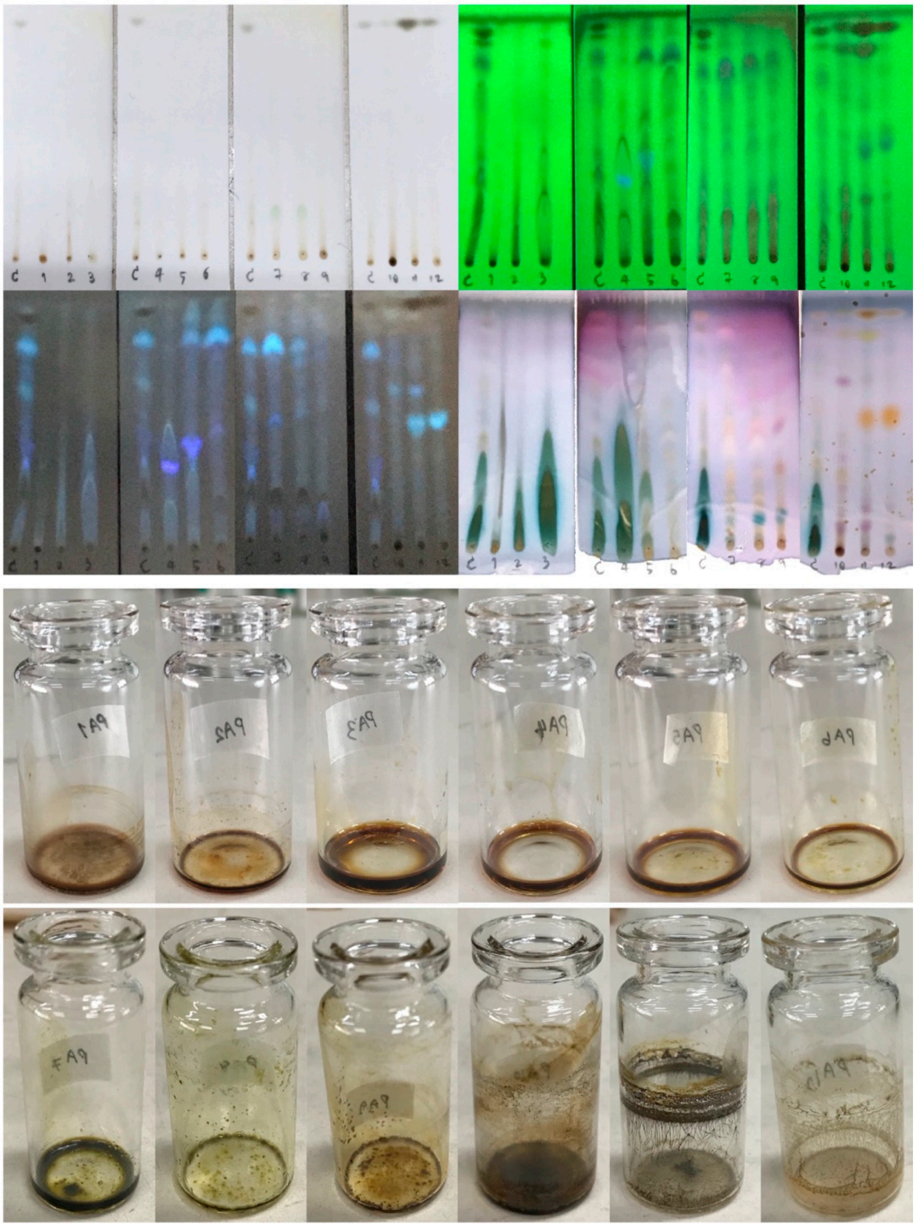

Figure S1. Characterization of fractions (PA1 to PA12) from the ethyl acetate extract. (A) Thin-layer chromatography plates: Fractions PA1-PA12 were visualized under daylight (upper left corner), short-wave ultraviolet light (upper right corner), long-wave ultraviolet light (lower left corner), and after spraying with *p*-anisaldehyde reagent (lower right corner). (B) Isolated fractions: Dry fractions PA1-PA12 are shown from top to bottom, left to right.

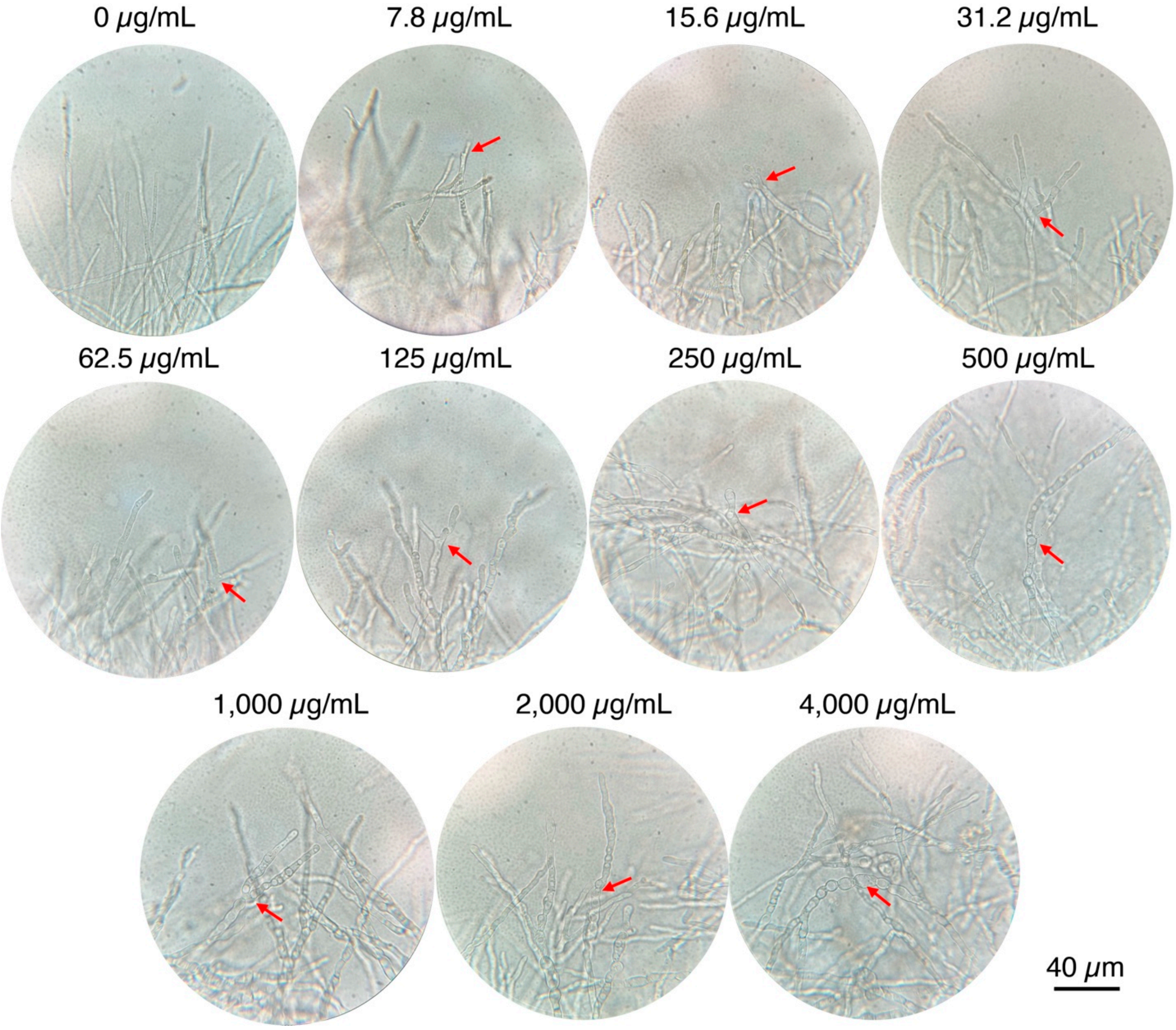

Figure S2. Characteristics of mycelia germinated from PA3-treated Col conidia (1,000X). The concentrations range from 4,000 to 7.8 μg/mL, with 0 μg/mL as the negative control. Red arrows indicate the observed abnormalities.

(A)

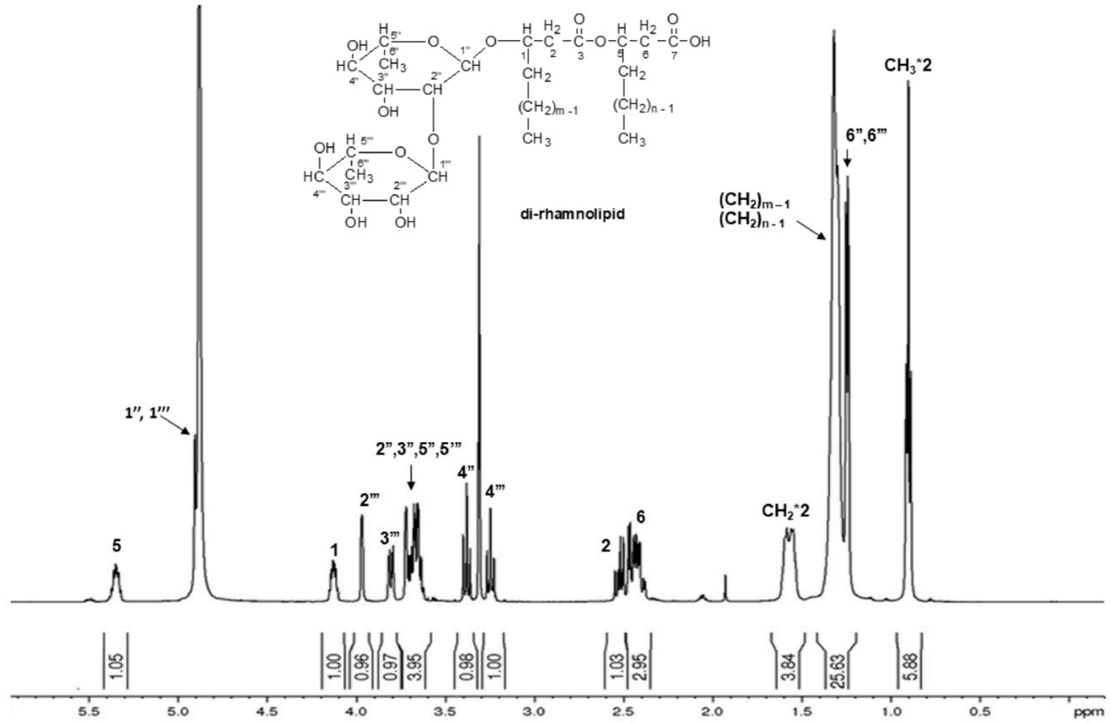

(B)

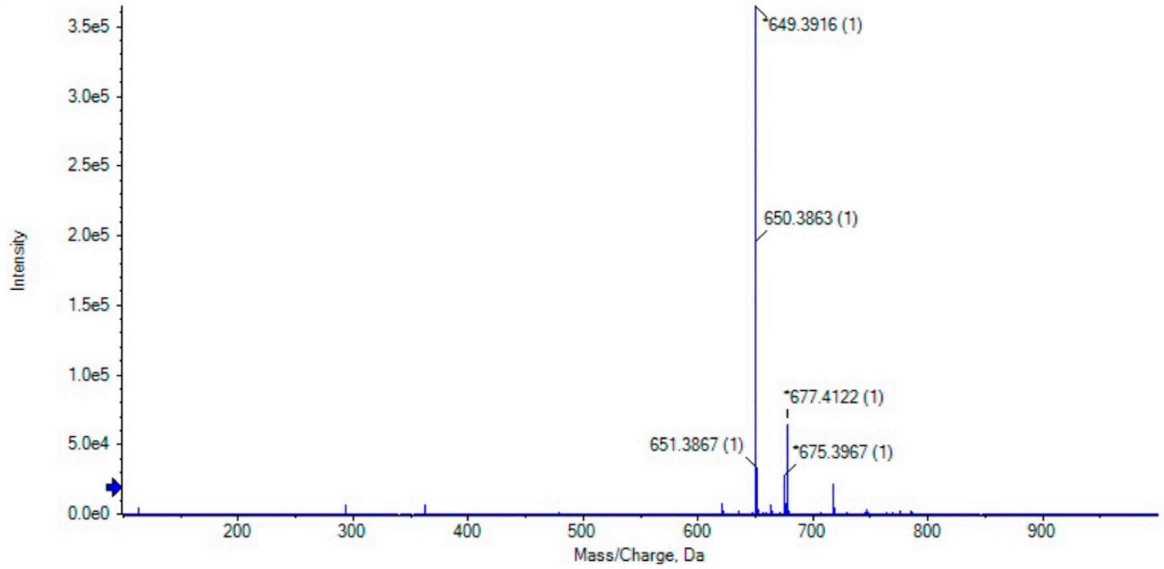

Figure S3. Characterization of PA3-1 by ^{1}H-NMR and ES-MS spectroscopic data. (A) ^{1}H-NMR spectrum of PA3-1 in CD_3OD. (B) Negative ion electrospray mass spectrum of PA3-1.

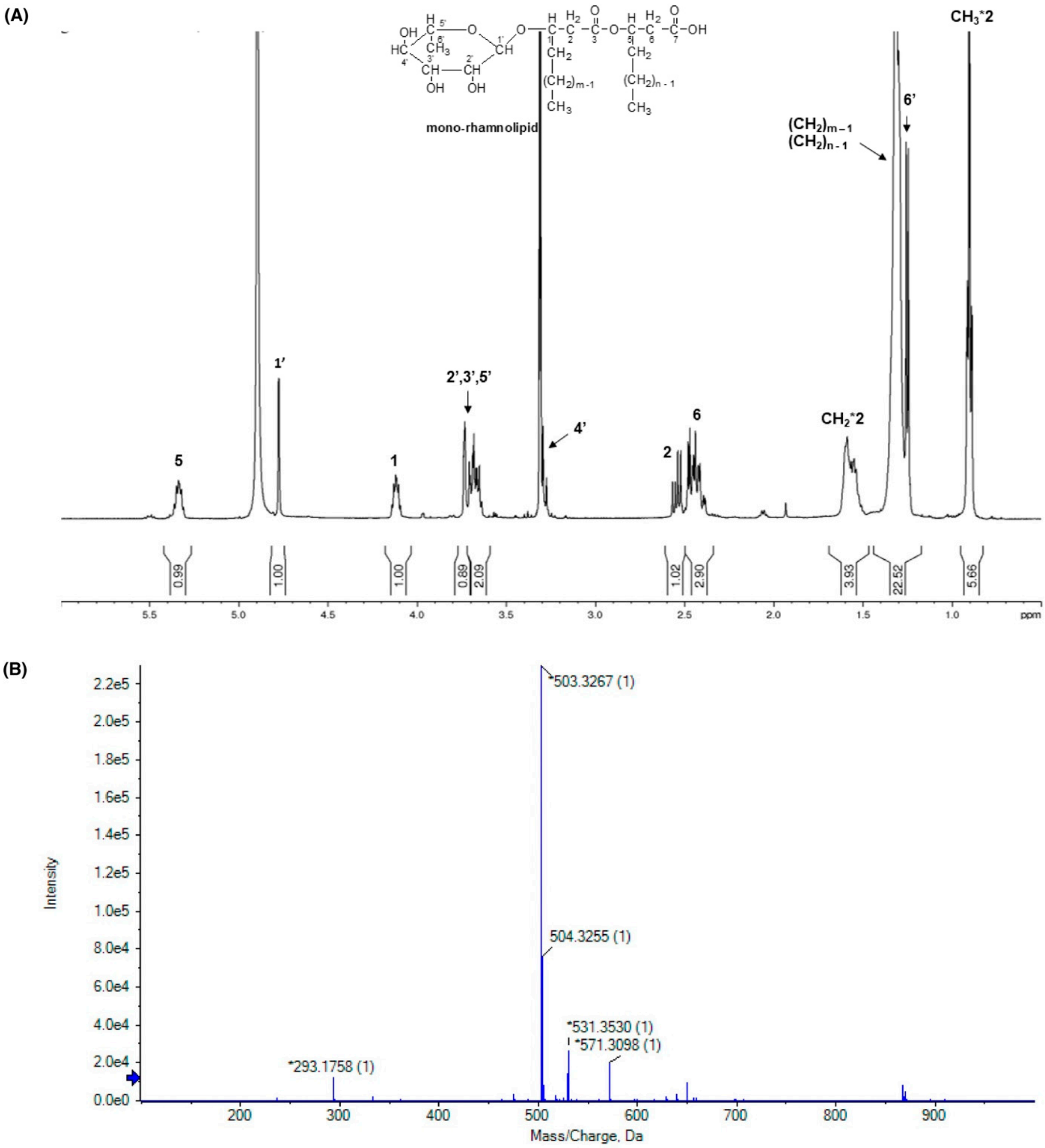

Figure S4. Characterization of PA3-2 by ^{1}H-NMR and ES-MS spectroscopic data. (A) ^{1}H-NMR spectrum of PA3-2 in CD_3OD. (B) Negative ion electrospray mass spectrum of PA3-2.

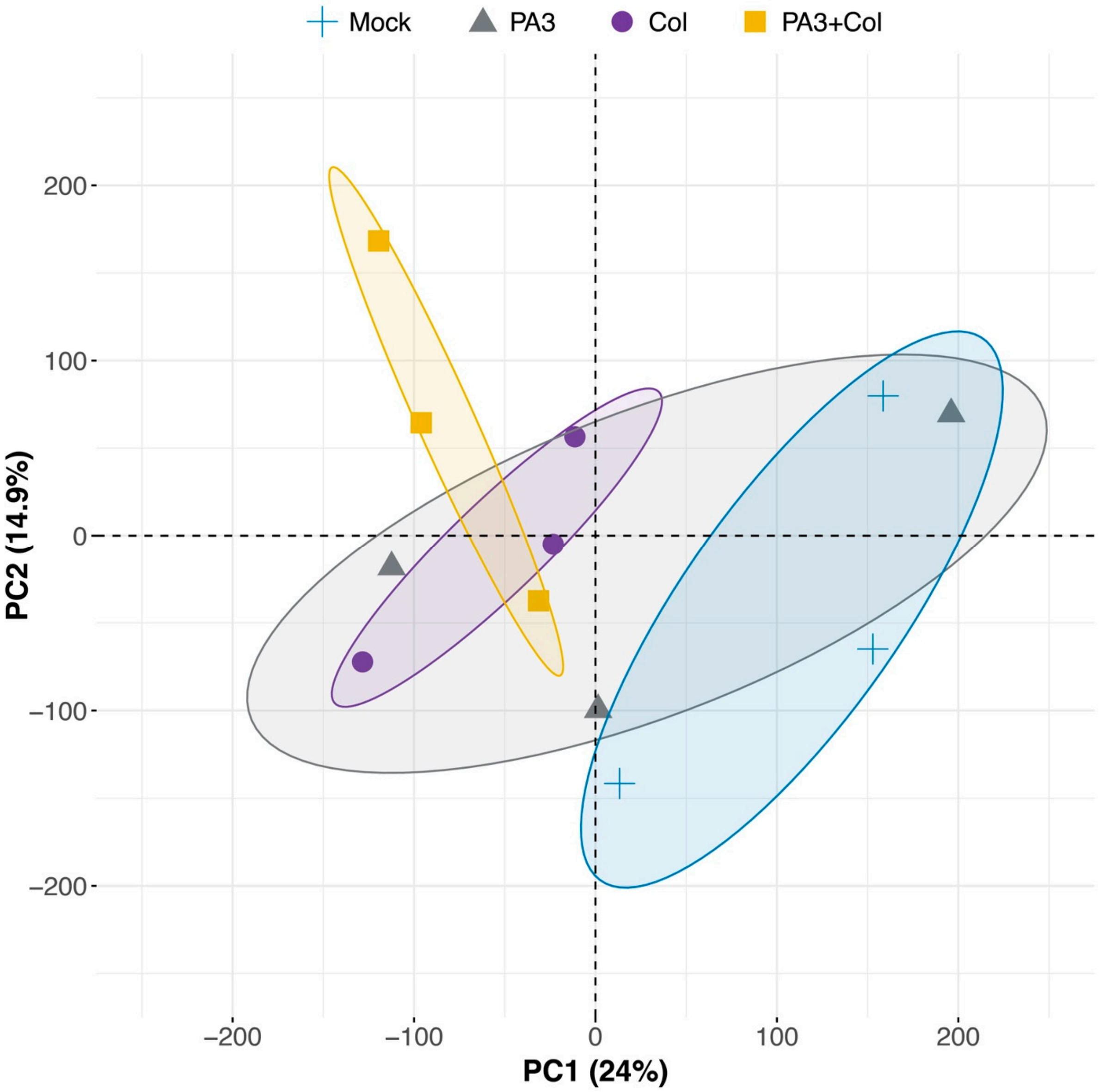

Figure S5. Principal component analysis (PCA) plot of normalized gene expression data, illustrating the variation between Mock (blue cross), PA3 (gray triangle), Col (purple circle), and PA3+Col (yellow square) treatments.

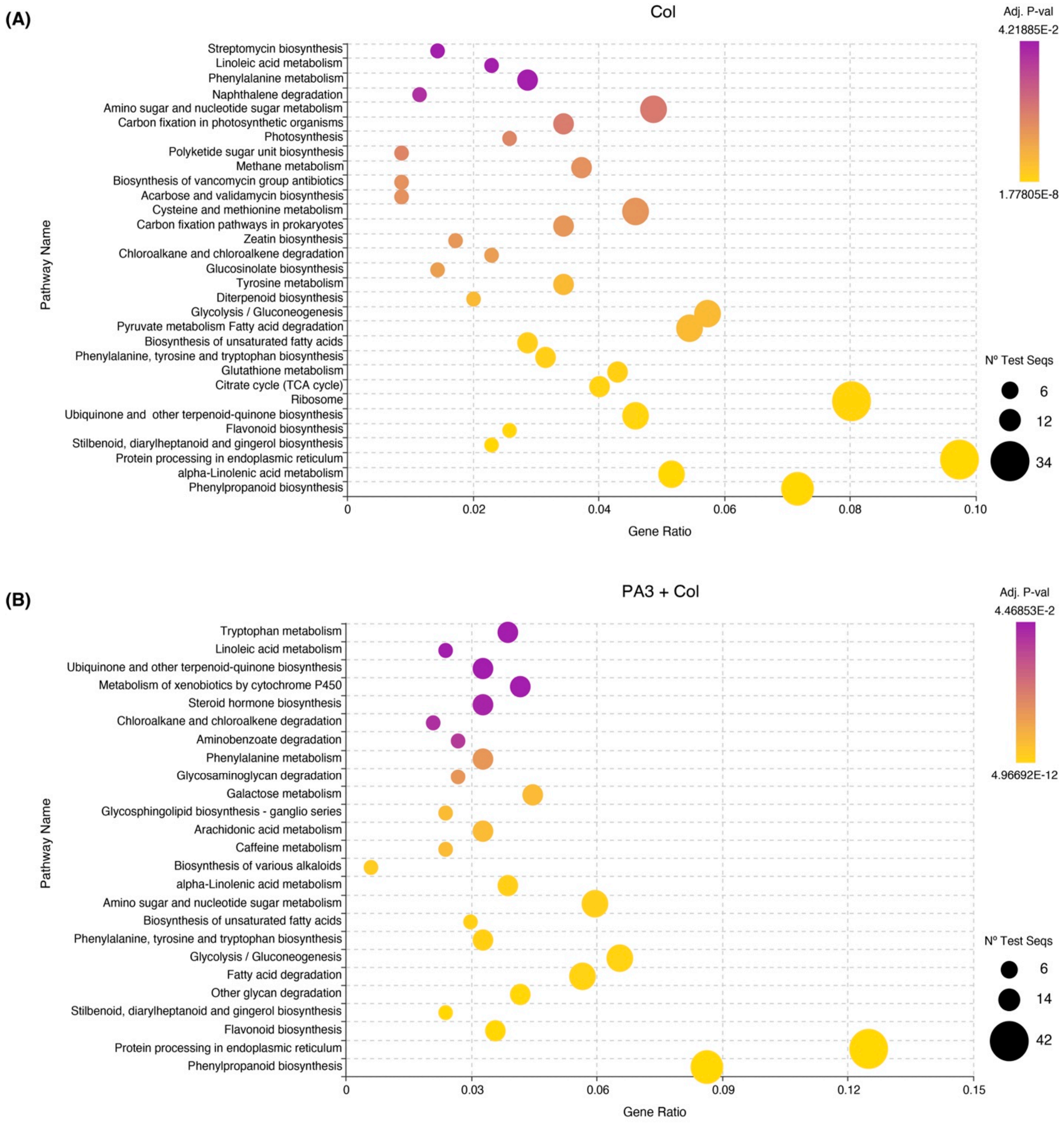

Figure S6. KEGG pathway enrichment analysis of DEGs. (A) Enriched pathways in the Col treatment alone. (B) Enriched pathways in the PA3 + Col treatment.
